# Comparative Genomic Study of Streptococcus anginosus Reveals Distinct Group of Urinary Strains

**DOI:** 10.1128/msphere.00687-22

**Published:** 2023-02-07

**Authors:** Ananya Prasad, Adriana Ene, Sandra Jablonska, Jingjie Du, Alan J. Wolfe, Catherine Putonti

**Affiliations:** a School of Biological Sciences, University of California San Diego, San Diego, California, USA; b Bioinformatics Program, Loyola University Chicago, Chicago, Illinois, USA; c Department of Microbiology and Immunology, Loyola University Chicago, Maywood, Illinois, USA; d Department of Biology, Loyola University Chicago, Chicago, Illinois, USA; University of Michigan—Ann Arbor

**Keywords:** *Streptococcus*, *Streptococcus anginosus*, urinary tract, urobiome

## Abstract

Streptococcus anginosus is a prevalent member of the human flora. While it has been found in the microbiota of “healthy” asymptomatic individuals, it has also been associated with genitourinary tract infections and bacteremia. Based upon multilocus sequence analysis, two subspecies and two genomosubspecies have been characterized for the species. We previously conducted whole-genome sequencing of 85 *S. anginosus* isolates from the urinary tract. Here, we present genomic analysis of this species, including isolates from the urinary tract as well as gut and fecal, vaginal, oral, respiratory, and blood and heart samples. Average nucleotide identity and core genome analysis revealed that these strains form two distinct groups. Group 1 is comprised of the *S. anginosus* type strain and other previously identified *S. anginosus* subspecies and genomosubspecies, including isolates from throughout the human body. In contrast, group 2 consists of predominantly urinary streptococci (*n* = 77; 85.6%). Both of these *S. anginosus* groups are distinct from other members of the Streptococcus anginosus group (SAG) species S. intermedius and S. constellatus. Genes conserved among all strains of one group but not in any strains in the other group were next identified. Group 1 strains included genes found in S. intermedius and *S. constellatus*, suggesting that they were lost within the ancestor of the group 2 strains. In contrast, genes unique to the group 2 strains were homologous to more distant streptococci, indicative of acquisition via horizontal gene transfer. These genes are ideal candidates for use as marker genes to distinguish between the two groups in the human microbiota.

**IMPORTANCE** Whole-genome analysis of *S. anginosus* strains provides greater insight into the diversity of this species than from marker genes alone. Our investigation of 166 publicly available *S. anginosus* genomes via average nucleotide identity and core genome analysis revealed two phylogenomically distinct groups of this species, with one group almost exclusively consisting of isolates from the urinary tract. In contrast, only 8 urinary strains were identified within the other group, which contained the *S. anginosus* type strain, as well as all identified subspecies and genomosubspecies. While genomic analysis suggested that this urinary group of *S. anginosus* is genomically different from the previously characterized *S. anginosus* subspecies, phenotypic characterization is still needed. Given prior reports of the prevalence of *S. anginosus* in the urinary tract of both continent and incontinent females, future studies are needed to investigate if the symptom state of the urinary tract is associated with these two different groups.

## OBSERVATION

Streptococcus anginosus is a prevalent member of the human flora, colonizing the oral cavity, upper respiratory tract, gastrointestinal tract, and female urogenital tract (1, 2). While long thought to be a commensal species, reports of *S. anginous* bacterial infections have increased over the last few decades (3–5). In most cases, these infections resulted in hospitalizations. A recent study found that *S. anginosus* was more abundant in the vaginal microbiota of postpartum females, and it has been associated with placental inflammation and chorioamnionitis (6, 7). Our prior studies of the female bladder microbiota found *S. anginosus* associated with urge urinary incontinence (8, 9), although it has also been routinely detected in continent controls (10). It also has been associated with genitourinary tract infections and bacteremia (for review, see reference 11). Genome analyses of *S. anginosus* isolates have found Streptococcus pyogenes virulence factor genes (12).

Earlier genomic analysis was instrumental in distinguishing the three species of the Streptococcus anginosus group (SAG): *S. anginosus*, S. constellatus, and S. intermedius (13). Complementing this genomic work, multilocus sequence analysis (MLSA) of *S. anginosus* strains has provided greater resolution of this species. Based on MLSA, two subspecies and two genomosubspecies have been identified: *S. anginosus* subsp. *whileyi*, *S. anginosus* subsp. *anginosus*, *S. anginosus* genomosubsp. AJ1, and *S. anginosus* genomosubsp. *vellorensis* (12, 14). While a previous pangenome analysis of the SAG genomes (*n* = 18, including 6 representatives of *S. anginosus*) has been reported (13), a large-scale pangenome analysis of just *S. anginosus* strains has yet to be conducted.

**FIG 1:**
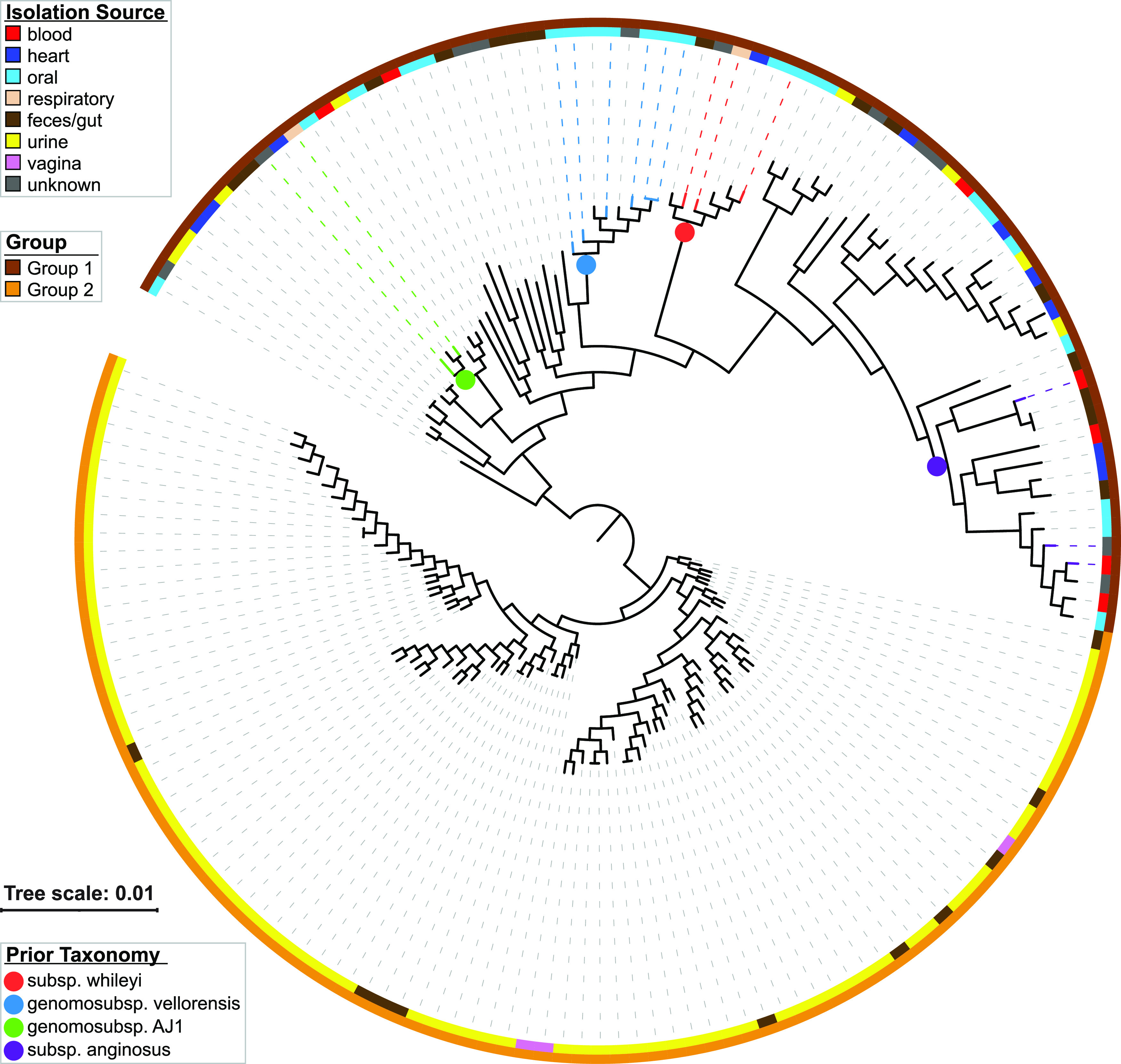
Phylogenetic tree of the *S. anginosus* core genome. The inner ring corresponds to the isolation source. The outer ring corresponds to the grouping defined here. Isolates associated with one of the *S. anginosus* genomosubspecies or subspecies are indicated by a colored branch as well as the dotted line connecting the branch to the isolation source and group ring. These designations were determined by referring to Babbar et al. (12).

All publicly available *S. anginosus* strains were retrieved from NCBI’s Assembly database and assessed for completeness and contamination using checkM (15). In total, 166 genome assemblies were considered further; their isolation source was retrieved from BioSample metadata and/or associated literature (see Table S1 in the supplemental material). Anvi’o v. 7.2 was used to annotate and identify the pangenome (16). The core genome of single-copy genes was identified (*n* = 532), and the aligned amino acid sequences of this core were retrieved from the Anvi’o pangenome database. A phylogenetic tree was derived using FastTree v.2.1.11 (17) through Geneious Prime v.2022.2.1 and visualized with iTOL v. 6.5.8 (18) (Fig. 1). Two distinct groups were identified, one containing the *S. anginosus* type strain and other previously identified *S. anginosus* subspecies (hereafter referred to as *S. anginosus* group 1) and the other containing predominantly urinary streptococci (*S. anginosus* group 2).

10.1128/msphere.00687-22.1 TABLE S1 Genome and isolation details regarding the *S. anginosus* strains included in this study. Download Table S1, XLSX file, 0.01 MB. Copyright © 2023 Prasad et al. 2023 Prasad et al. https://creativecommons.org/licenses/by/4.0/ This content is distributed under the terms of the Creative Commons Attribution 4.0 International license.

As Fig. 1 shows, 77 of the 90 strains in *S. anginosus* group 2 were isolated from the urinary tract. Three were isolated from vaginal samples, and 10 were isolated from fecal or gut samples. Two of the vaginal isolates were from vaginal swabs from females with no clinical genitourinary symptoms (19); the other vaginal strain was isolated from vaginal fluid collected from a pregnant woman diagnosed with bacterial vaginosis (20). With regard to the fecal and gut samples, three of the genomes were metagenome-assembled genomes (21–23). The others were isolates from stool samples (24, 25). Only 8 isolates from urine were found in *S. anginosus* group 1, which also includes strains isolated from blood, heart, respiratory, oral, and fecal samples.

**FIG 2:**
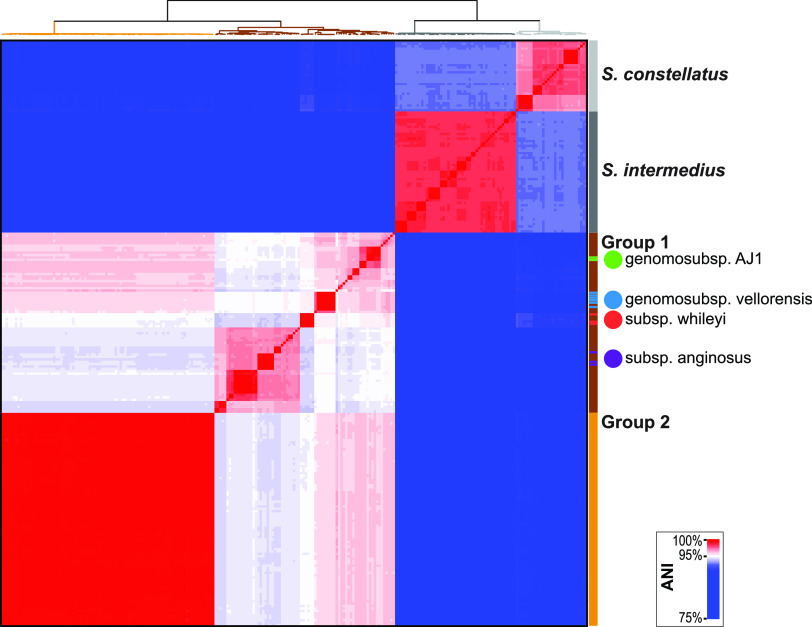
ANI analysis of publicly available genome assemblies of members of *S. anginosus* strains and *S. constellatus* and S. intermedius. The bar on the right indicates the species, group, and genomosubspecies or subspecies. ANI values of >95% are shown in red hues; ANIs of <95% are shown in blue hues. The tree at the top of the heatmap indicates the ANI-based relationship of the sequences examined, colored by species or group.

Because average nucleotide identity (ANI) is the commonly used metric to delineate species, we next computed the ANI using PyANI v.0.2.11 (26). For this calculation, we also included publicly available strains of other members of SAG: S. intermedius (*n* = 51) and *S. constellatus* (*n* = 30) (Table S2). ANI analysis confirmed that both *S. anginosus* groups were distinct from S. intermedius and *S. constellatus* (Fig. 2; Table S3). Furthermore, the ANI values enabled us to associate additional strains with *S. anginosus* subsp. *whileyi* and the two genomosubspecies, which are listed in Table S1. This analysis also showed that group 2 genomes were more similar to the genomes of the *S. anginosus* genomosubsp. AJ1 and *S. anginosus* genomosubsp. *vellorensis* strains than they were to the *S. anginosus* subsp. *whileyi* and *S. anginosus* subsp. *anginosus* strains (Table S3). When compared to strains assigned to these two genomosubspecies, the group 2 strains had an ANI value of 95.66%. This slightly exceeded the 95% threshold commonly used to distinguish species (27). In contrast, none of the group 2 strains had an ANI value greater than the 95% threshold to any of the examined group 1 *S. anginosus* subsp. *whileyi* or *S. anginosus* subsp. *anginosus* isolates. The ANI-based clustering of the strains examined identified four main branches (Fig. 2). From left to right, the group 2 *S. anginosus* strains, the group 1 *S. anginosus* strains (including *S. anginosus* subsp. *anginosus*, *S. anginosus* subsp. *whileyi*, *S. anginosus* genomosubsp. *vellorensis*, and *S. anginosus* genomosubsp. AJ1), the S. intermedius strains, and the *S. constellatus* strains are shown.

10.1128/msphere.00687-22.2 TABLE S2 S. intermedius and *S. constellatus* genome assemblies included in the ANI comparison to *S. anginosus* strains. Download Table S2, XLSX file, 0.01 MB. Copyright © 2023 Prasad et al. 2023 Prasad et al. https://creativecommons.org/licenses/by/4.0/ This content is distributed under the terms of the Creative Commons Attribution 4.0 International license.

10.1128/msphere.00687-22.3 TABLE S3 ANI ranges (minimum to maximum) between *S. constellatus* (SC), S. intermedius (SI), strains grouped in the genomosubspecies (Group_1_genomosubsp), strains grouped in the subspecies *whileyi* (Group_1_subspW), strains grouped in the subspecies *anginosus* (Group_1), and Group 2 strains (Group_2). (Values in parentheses are average ANI values). Download Table S3, DOCX file, 0.01 MB. Copyright © 2023 Prasad et al. 2023 Prasad et al. https://creativecommons.org/licenses/by/4.0/ This content is distributed under the terms of the Creative Commons Attribution 4.0 International license.

To further explore the genetic differences between the *S. anginosus* group 1 and group 2 strains, we next identified genes conserved among all strains of one group but not in any strains in the other group. Group 1 included 1,040 genes conserved among all 76 of its strains. Only 10 of these genes, however, were unique to this group (i.e., they were not present in any of the assemblies from group 2). Querying the amino acid sequences of these 10 genes against the complete nr database revealed these genes to be conserved among *S. constellatus* and S. intermedius strains (Table S4). The first seven proteins listed in Table S4 are contiguous, suggesting possible horizontal gene transfer from other species of SAG, namely, *S. constellatus* and/or S. intermedius, to the common ancestor of the group 1 strains or loss in the common ancestor of the group 2 strains. Group 2 strains had 1,393 genes conserved among all 90 strains. Ten of these genes were not found in any of the group 1 strains. When queried against the complete nr database, 3 of these sequences did not have significant sequence similarity to any records. The other protein sequences, however, had homologs in Streptococcus gallolyticus and Streptococcus pantholopis strains and/or strains from the S. mitis/*oralis* group (Table S5). The first six genes listed in Table S5 are contiguous. The contiguous group-specific genes were likely acquired via horizontal gene transfer. Prior studies have shown that the genus is naturally competent (28), and gene exchange occurs between Streptococcus species (29); this supports the hypothesis that the contiguous group 1- or group 2-specific genes may have been acquired via horizontal gene transfer. Further investigation into the genes unique to group 2 is needed to ascertain if they benefit the bacterium in the urinary tract environment.

10.1128/msphere.00687-22.4 TABLE S4 Details of gene sequences conserved among all group 1 strains that were not present in any of the group 2 strains. (*Only hits with query coverage and sequence identity of ≥85% were reported. Taxonomic names reported are according to the “Organism” designation in the GenBank records of hits). Download Table S4, DOCX file, 0.01 MB. Copyright © 2023 Prasad et al. 2023 Prasad et al. https://creativecommons.org/licenses/by/4.0/ This content is distributed under the terms of the Creative Commons Attribution 4.0 International license.

10.1128/msphere.00687-22.5 TABLE S5 Details of gene sequences conserved among all of the group 2 strains that were not present in any of the group 1 strains. (*Only hits with query coverage and sequence identity of ≥85% are reported. Taxonomic names reported are according to the “Organism” designation in the GenBank records of hits). Download Table S5, DOCX file, 0.01 MB. Copyright © 2023 Prasad et al. 2023 Prasad et al. https://creativecommons.org/licenses/by/4.0/ This content is distributed under the terms of the Creative Commons Attribution 4.0 International license.

The majority (91%) of the urinary isolate genomes were assigned to group 2. The group 2 strains have ANI values of <95% with all strains of both *S. anginosus* subspecies (*n* = 42), and the core genome analysis further supported the distinction between the group 1 and group 2 genomes. However, the group 2 genomes had ANI values of >95% with the two genomosubspecies (*n* = 34). Distinct phenotypic characteristics for both of these genomosubspecies have yet to be identified or investigated (12, 14). Phenotypic characterization of the group 2 strains also is needed to ascertain if this is a new species or subspecies within the SAG. Given prior reports of the prevalence of *S. anginosus* in the urinary tract of both continent and incontinent females (8–10), it would be interesting to see if continence and incontinence are associated with these two different groups. Among the 85 *S. anginosus* isolates from our own collection, we note that isolates from females without lower urinary tract symptoms are only represented in the group 2 strains (*n* = 10) (Table S1). However, group 2 strains also include isolates from females with urinary tract infection, recurrent urinary tract infection, and incontinence. Thus, further isolation of *S. anginosus* strains from females without lower urinary tract symptoms is needed. The unique genes identified here could serve as marker genes to distinguish between the two groups.
