# Supplementary material for: Comparative Genomic Study of Streptococcus anginosus Reveals Distinct Group of Urinary Strains
Source: mSphere. 2023 Feb 7;8(2):e00687-22. doi: 10.1128/msphere.00687-22 (PMC10117062; doi:10.1128/msphere.00687-22)
Supplement: TABLE S3 [file msphere.00687-22-s0003.docx]

**Table S3.** ANI ranges (minimum – maximum) between *S. constellatus* (SC), *S. intermedius* (SI), strains grouped in the genomosubspecies (Group_1_genomosubsp), strains grouped in the subspecies *whileyi* (Group_1_subspW), strains grouped in the subspecies *anginosus* (Group_1), and Group 2 strains (Group_2). In the parentheses, the average ANI is reported.

|  | **SC** | **SI** | **Group_1_**  **genomosubsp** | **Group_1_**  **subspW** | **Group_1** | **Group_2** |
| --- | --- | --- | --- | --- | --- | --- |
| SC | 96.15-100 (97.63) | 91.91-92.61 (92.24) | 88.98-90.71 (89.85) | 90.15-91.72 (90.62) | 88.35-89.97 (89.17) | 89.23-90.44 (89.67) |
| SI | 91.91-92.61 (92.24) | 98.1-100 (98.45) | 87.67-88.56 (88.09) | 88.8-89.34 (88.98) | 87.48-88.44 (87.84) | 87.71-88.55 (87.98) |
| Group_1_genomosubsp | 88.98-90.71 (89.85) | 87.67-88.56 (88.09) | 94.72-100 (96.25) | 94.25-95.48 (95.02) | 93.66-95.14 (94.53) | 95.24-96.17 (95.66) |
| Group_1_subspW | 90.15-91.72 (90.62) | 88.8-89.34 (88.98) | 94.25-95.48 (95.02) | 99.37-99.97 (99.58) | 93.62-94.23 (93.88) | 94.34-94.85 (94.62) |
| Group_1 | 88.35-89.97 (89.17) | 87.48-88.44 (87.84) | 93.66-95.14 (94.53) | 93.62-94.23 (93.88) | 95.62-100 (97.1) | 93.81-94.68 (94.24) |
| Group_2 | 89.23-90.44 (89.67) | 87.71-88.55 (87.98) | 95.24-96.17 (95.66) | 94.34-94.85 (94.62) | 93.81-94.68 (94.24) | 99.15-100 (99.68) |
