# Supplementary material for: Comparative Genomic Study of Streptococcus anginosus Reveals Distinct Group of Urinary Strains
Source: mSphere. 2023 Feb 7;8(2):e00687-22. doi: 10.1128/msphere.00687-22 (PMC10117062; doi:10.1128/msphere.00687-22)
Supplement: TABLE S4 [file msphere.00687-22-s0004.docx]

**Table S4.** Details of gene sequences conserved among all Group 1 strains that are not present in any of the Group 2 strains. *Only hits with query coverage and sequence identity ≥ 85% are reported. Taxonomic names reported are according to the “Organism” designation in the GenBank records of hits.

| **Gene Cluster ID** | **Predicted Function** | **Query Coverage/ Sequence Identity to Top Hit** | **Accession No. of Top Hit** | **Hits to Other Taxa (Query Coverage/ Sequence Identity)*** |
| --- | --- | --- | --- | --- |
| GC_00001776 | Thiamine biosynthesis protein ApbE | 100/100 | EJP25027.1 | *S. milleri* (100/97); *S. constellatus* (100/90); *S. intermedius* (100/86) |
| GC_00001754 | NADPH-dependent FMN reductase | 100/100 | KAA9296846.1 | *S. intermedius* (100/98); *S. milleri* (100/97); *S. periodonticum* (100/97); *S. massiliensis* (100/90); *S. oricebi* (100/89); *S. cristatus* (100/89); *S. panodentis* (100/87); *S. ratti* (100/86); *Abiotrophia defectiva* (100/85); *Granulicetella elegans* (100/85); *S. ursoris* (99/86); *S. mutans* (99/85); *S. troglodytae* (99/85) |
| GC_00001779 | NADPH-dependent FMN reductase | 100/100 | KUM00782.1 | *S. milleri* (100/99); *S. intermedius* (100/98); *S. periodonticum* (100/98); *S. parasanguinis* (99/85); *S. oricebi* (99/85); *S. oralis* (99/85) |
| GC_00001774 | ABC transporter, ATP-binding protein | 100/100 | KAA9296888.1 | *S. intermedius* (100/99); *S. periodonticum* (100/99); *S. constellatus* (100/98); *S. milleri* (100/97) |
| GC_00001783 | Putative ABC-2 family transporter | 100/100 | KAA9296848.1 | *S. milleri* (100/99); *S. intermedius* (100/99); *S. constellatus* (100/99); *S. periodonticum* (100/98) |
| GC_00001778 | Putative ABC-2 family transporter | 100/100 | KAA9296849.1 | *S. periodonticum* (100/99); *S. intermedius* (100/98); *S. constellatus* (100/98); *S. milleri* (100/98) |
| GC_00001766 | Sensor histidine kinase | 100/100 | KAA9296850.1 | *S. periodonticum* (100/98); *S. milleri* (100/97); *S. intermedius* (100/97) |
| GC_00001756 | Hypothetical protein | 100/100 | KAA9295420.1 | *S. periodonticum* (100/96); *S. constellatus* (100/92); *S. milleri* (100/91); *S. intermedius* (100/92) |
| GC_00001781 | Polyphosphate polymerase domain-containing protein | 100/99 | KAA9297767.1 | *S. milleri* (100/98); *S. constellatus* (100/97); *S. intermedius* (100/96) |
| GC_00001782 | FAD-containing oxidoreductase | 100/100 | KAA9296793.1 | *S. milleri* (100/97); *S. periodonticum* (100/96); *S. constellatus* (100/90); *S. intermedius* (100/90); *S. sanguinis* (100/85); *S. gordonii* (100/85) |
