# Supplementary material for: Comparative Genomic Study of Streptococcus anginosus Reveals Distinct Group of Urinary Strains
Source: mSphere. 2023 Feb 7;8(2):e00687-22. doi: 10.1128/msphere.00687-22 (PMC10117062; doi:10.1128/msphere.00687-22)
Supplement: TABLE S5 [file msphere.00687-22-s0005.docx]

**Table S5.** Details of gene sequences conserved among all of the Group 2 strains that are not present in any of the Group 1 strains. *Only hits with query coverage and sequence identity ≥ 85% are reported. Taxonomic names reported are according to the “Organism” designation in the GenBank records of hits.

| **Gene Cluster ID** | **Predicted Function** | **Query Coverage/ Sequence Identity to Top Hit** | **Accession No. of Top Hit** | **Hits to Other Taxa (Query Coverage/ Sequence Identity)*** |
| --- | --- | --- | --- | --- |
| GC_00001622 | 6-phospho 3-hexuloisomerase | 100/100 | ETI84922.1 | *Aerococcus sanguinicola* (100/99); *S. gallolyticus* (100/97) |
| GC_00001668 | GNAT family acetyltransferase | 100/100 | ETI84466.1 | *S. oralis* (100/96); *S. mitis* (100/94); *S. pneumoniae* (99/94); *S. pseudopneumoniae* (99/93); *S. cristatus* (100/93) |
| GC_00001661 | PTS galactitol transporter subunit IIB | 100/100 | ETI84924.1 | *S. gallolyticus* (100/98); *S. pantholopis* (97/92) |
| GC_00001646 | PTS galactitol transporter subunit IIC | 100/100 | ETI84925.1 | *S. gallolyticus* (100/98); *A. sanguinicola* (100/98); *S. pantholopis* (99/94); *E. durans* (99/87); *E. faecalis* (99/87) |
| GC_00001654 | RpiB (ribose 5-phosphate isomerase B) | 100/100 | ETI84926.1 | *S. gallolyticus* (100/97); *S. pantholopis* (100/92); *Enterococcus faecium* (100/86); *E. avium* (100/86); *E. faecalis* (100/85) |
| GC_00001671 | DeoR/GlpR transcriptional regulator | 100/100 | ETI84927.1 | *S. gallolyticus* (100/94); *S. pantholopis* (100/88) |
| GC_00001486 | Can B-type domain-containing protein | 100/99.73 | KAA9294504.1 | None |
| GC_00001666 | Hypothetical protein | 100/100 | ETI86902.1 | None |
| GC_00001660 | Hypothetical protein | 100/100 | ETI84257.1 | None |
| GC_00001663 | GNAT family acetyltransferase | 100/100 | ETI84466.1 | *S. oralis* (100/96); *S. mitis* (100/94); *S. pneumoniae* (99/94); *S. pseudopneumoniae* (99/93); *S. cristatus* (100/93) |
